# How "Community" Matters for How People Interact With Information: Mixed Methods Study of Young Men Who Have Sex With Other Men

**DOI:** 10.2196/jmir.2370

**Published:** 2013-02-21

**Authors:** Tiffany Christine Veinot, Chrysta Cathleen Meadowbrooke, Jimena Loveluck, Andrew Hickok, Jose Artruro Bauermeister

**Affiliations:** ^1^School of InformationUniversity of MichiganAnn Arbor, MIUnited States; ^2^Department of Health Behavior and Health EducationSchool of Public HealthUniversity of MichiganAnn Arbor, MIUnited States; ^3^HIV/AIDS Resource CenterYpsilanti, MIUnited States

**Keywords:** Community, health informatics, information use, information seeking, incidental information acquisition, relevance, social networks, HIV/AIDS, information sharing, mixed methods, consumer health informatics

## Abstract

**Background:**

We lack a systematic portrait of the relationship between community involvement and how people interact with information. Young men who have sex with men (YMSM) are a population for which these relationships are especially salient: their gay community involvement varies and their information technology use is high. YMSM under age 24 are also one of the US populations with the highest risk of HIV/AIDS.

**Objective:**

To develop, test, and refine a model of gay community involvement (GCI) factors in human-information interaction (HII) as applied to HIV/AIDS information among YMSM, specifically examining the role of Internet use in GCI and HII.

**Methods:**

Mixed methods included: 1) online questionnaire with 194 YMSM; and 2) qualitative interviews with 19 YMSM with high GCI levels. Recruitment utilized social media, dating websites, health clinics, bars/clubs, and public postings. The survey included questions regarding HIV/AIDS–related information acquisition and use patterns, gay community involvement, risk behaviors, and technology use. For survey data, we tested multiple linear regression models using a series of community- and information-related variables as dependent variables. Independent variables included community- and information-related variables and demographic covariates. We then conducted a recursive path analysis in order to estimate a final model, which we refined through a grounded theory analysis of qualitative interview data.

**Results:**

Four community-related variables significantly predicted how people interact with information (HII variables): 1) gay community involvement (GCI), 2) social costs of information seeking, 3) network expertise accessibility, and 4) community relevance. GCI was associated with significantly lower perceived social costs of HIV/AIDS information seeking (*R* ^*2*^=0.07). GCI and social costs significantly predicted network expertise accessibility (*R* ^*2*^=0.14). GCI predicted 14% of the variance in community relevance and 9% of the variance in information seeking frequency. Incidental HIV/AIDS information acquisition (IIA) was also significantly predicted by GCI (*R* ^*2*^=0.16). 28% of the variance in HIV/AIDS information use was explained by community relevance, network expertise access, and both IIA and information seeking. The final path model showed good fit: the RSMEA was 0.054 (90% CI: .000-.101); the Chi-square was non-significant (χ^2^(11)=17.105; *P*=.105); and the CFI was 0.967. Qualitative findings suggest that the model may be enhanced by including information sharing: organizing events, disseminating messages, encouraging safety, and referring and recommending. Information sharing emerged under conditions of pro-social community value enactment and may have consequences for further HII. YMSM with greater GCI generally used the Internet more, although they chatted online less.

**Conclusions:**

HIV/AIDS–related HII and associated technology uses are community-embedded processes. The model provides theoretical mediators that may serve as a focus for intervention: 1) valuing HIV/AIDS information, through believing it is relevant to one’s group, and 2) supportive and knowledgeable network members with whom to talk about HIV/AIDS. Pro-social community value endorsement and information sharing may also be important theoretical mediators. Our model could open possibilities for considering how informatics interventions can also be designed as community-level interventions and vice versa.

## Introduction

Experts increasingly recognize that human-information interaction (HII)—including acquisition, sharing, management, and use of information—is a social phenomenon. A host of research approaches have shed light on this social character, from interactionism to network analysis [1]. This socially oriented research has provided several valuable insights regarding HII in human communities, including the possibility of information technology (IT) use to establish and reinforce community identities, and the potential of IT deployed in geographic communities to shift the nature and extent of ties between residents [1]. We have also learned that the situational relevance of information varies by community, leading to selective information acceptance in different groups [2]. Additionally, Chatman’s “Theory of Information Poverty” tells us that social costs associated with seeking certain forms of information within a community may result in information avoidance [2,3]. Despite these observations, we lack a systematic portrait of the relationship between people’s everyday community involvements and their HII, including *how important* community involvement might be in the emergence of these patterns [1,4]. Moreover, there has been little dialogue between the fields of community informatics and health informatics, despite growing interest in embedding social influence in consumer health informatics (CHI) applications [5]. Therefore, our objective in this paper is to develop, test, and refine a model of community involvement factors in HII, as applied to the specific situation of HIV/AIDS among young men who have sex with men (YMSM) aged 18-24. Young men offer a particularly salient population in which to examine the relationship between community involvement and HII, since their gay community involvement varies [6], and their use of information technologies is high [7,8]. With a goal of informing HIV/AIDS prevention, we also focused on YMSM because they are one of the highest HIV/AIDS risk groups in United States [9].

The health domain offers a critical context in which to understand the role of community involvement in HII. Disease prevalence, incidence, and outcomes may all vary at a group level. In the case of HIV/AIDS in the United States, men who have sex with men (MSM) have long had disproportionately high rates of this disease, with the rate of new infections particularly high among African-American MSM [10-12], as well as those under age 24 [9]. Not all MSM identify as gay or bisexual; thus, public health practitioners created the term “men who have sex with men” to highlight the fact that many men who engage in same-gender sexual contact do not identify as gay or bisexual, although they may be behaviorally at risk for HIV infection [13]. Flores and colleagues [14] distinguish between identity, which they call the “self-view” of sexual identity, and community involvement, which they call the “social-normative view.” The social-normative view reflects one’s social and psychological connection to the gay community, which in turn affects the extent to which a person is influenced by that group. Even among people who identify as gay or bisexual, gay community involvement—factors such as socializing with other MSM or participating in lesbian, gay, bisexual and transgendered (LGBT) organizations—varies. Notably, non-gay *identification* among MSM may be particularly common among African Americans and Latinos [15,16]; however, gay community *involvement* varies less by race than *identity* does [14]. Understanding an MSM’s extent of gay community involvement may be particularly relevant to HII because it refers to connection to the group rather than internal perceptions of self. Thus, we outline below a series of hypotheses about potential relationships between these two aspects of behavior in our study population.

Due to the historical and present burden of HIV/AIDS among MSM, gay communities have mobilized an unprecedented response to the disease. Indeed, gay communities led the formation of many organizations and publications that develop and disseminate information about HIV/AIDS prevention and treatment [17-19]. Gay community settings are also frequently the focus of HIV/AIDS prevention efforts (eg, [20-22]). Due to the high prevalence of HIV/AIDS among MSM, members of this population may also be more likely to know people with HIV/AIDS (PHAs)—a social network factor associated with talking more and knowing more about the disease [23]. Thus, even though gay communities are not devoid of HIV-related stigmatization [24], we contend that YMSM who are more involved in the gay community will experience greater exposure to positive attitudes towards PHAs that circulate among some gay community segments and that they will also be exposed more to norms that support acknowledging and responding to personal risk for HIV/AIDS. Therefore, we hypothesize that:

H1: YMSM who are more involved in the gay community will report fewer perceived social costs of HIV/AIDS–related information seeking.

Kippax et al argued more than 20 years ago that MSM who are more involved in the gay community have more access to “informed social support” [25]. This thesis suggests a greater tendency for a man’s close associates to discuss HIV/AIDS with him and for these associates to be knowledgeable about the disease. Therefore, we hypothesize that:

H2: YMSM who are more involved in the gay community will have greater network access to HIV/AIDS expertise. Those who perceive fewer social costs of seeking HIV/AIDS information will also have more of this access.

A belief that HIV/AIDS is relevant to one’s community may also be a consequence of HIV/AIDS prevention efforts and personal acquaintance with PHAs. Moreover, people may be more likely to look for information that is perceived as relevant to their community [2]. Hence, we posit that:

H3: YMSM who are more involved in the gay community will believe that HIV is more relevant to their community.

Young MSM who are more involved in the gay community may frequently encounter an HIV/AIDS information-rich environment [26] and thus may be more frequently “exposed” [27] to such information “incidentally” [28] through people, documents, or the Internet. Thus, YMSM who are more involved in the gay community may also be more likely to have been exposed to HIV prevention messages and testing through venues such as bars, events, and gay websites [29]. Exposure to HIV/AIDS information through public health campaigns is also associated with supplementary information seeking [30]. Furthermore, knowing a PHA may give rise to more “network-mediated opportunities”—socially comfortable opportunities for asking questions about HIV/AIDS [27]. Prior research conducted in rural Canada also shows that higher levels of HIV/AIDS-related expertise and resources in a community may predict information acquisition success among its members [1]. Therefore, we hypothesize that:

H4: YMSM who are more involved in the gay community will report more incidental acquisition of HIV/AIDS information.

H5: YMSM who are more involved in the gay community will report more HIV/AIDS–related information seeking. People who perceive fewer social costs of HIV/AIDS information seeking, who see the disease as more relevant to their community, and who obtain HIV/AIDS information incidentally more often will also seek this information with more frequency.

People do not use all the information to which they have access. What factors determine information use? Certainly, information must be acquired before it is used. However, information provided by strong network ties [31] or perceived as collectively relevant may be more likely to be used [2]. Therefore, we contend:

H6: YMSMs’ use of HIV/AIDS information will be predicted by greater gay community involvement, higher levels of HIV/AIDS information acquisition (seeking information, incidental exposure), greater perceived relevance of HIV/AIDS to one’s community, and more network access to HIV/AIDS information (“network expertise accessibility”).

### A Model of Community Involvement Factors in Human-Information Interaction

**Figure 1:**
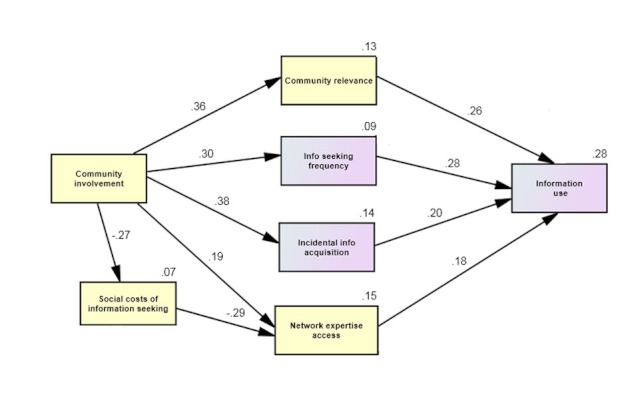
Model of community involvement factors in human-information interaction.

In addition to testing these hypotheses separately, we estimate a model (Figure 1) that considers each of these community involvement factors simultaneously. This model allows us to test the possibility of mediating effects of community factors in HII, while comparing the relative importance of these factors. Our model posits that gay community involvement will exert both direct and indirect effects on information acquisition and use. Thus, we forward the following hypotheses concerning indirect effects:

H7: Community involvement will exert indirect effects on information seeking through its effect on social costs of information seeking and community relevance.

H8: Community involvement will exert indirect effects on information use through its influence on information acquisition, perceived community relevance, and network expertise accessibility.

#### Model Refinement

Finally, through an inductive portion of the research, we assess the potential for new community involvement-related variables to explain the dependent variables included in the model. Therefore, we pose the following research question:

RQ1: What additional gay community-related factors, if any, may help to explain HIV/AIDS–related HII among YMSM?

### Technology and Community

Although each of the above HII processes may involve technologies, a focus on health informatics draws our attention to the extent of technological mediation of MSM’s gay community involvements and HII. According to studies, Internet use may be fundamentally changing gay communities in western countries (eg, [32,33]). Gay bars and other face-to-face settings are increasingly supplanted by use of the Internet to meet sex partners [34,35], sparking efforts to develop and test online HIV prevention initiatives [36]. MSM, including youth who may lack offline gay/bisexual associates, also report frequenting gay websites to meet friends [37,38]. The Internet may also facilitate offline community involvement by connecting people to gay groups and events [39]. In addition, advocates of a range of causes increasingly engage in online social activism [40]. Given these observations, we hypothesize that:

H9: YMSM who are more involved in the gay community will use technologies to socialize with others more, as well as to acquire HIV/AIDS information online more than YMSM who are less involved.

## Methods

### Mixed Methods Study

As part of a larger investigation of HIV testing among YMSM, we conducted a parallel, mixed methods study [41] including: 1) an online survey and 2) qualitative, in-depth, individual interviews. Eligibility criteria included identifying as a man who has had sex with other men in the past 6 months, being age 18 to 24, and living in southeastern Michigan. To further our goal of informing HIV/AIDS prevention, the research focused on YMSM in this age group due to the alarming 22% increase in new HIV infections in MSM under age 24 between 2008 and 2010 [9]. We obtained a Certificate of Confidentiality from the National Institutes of Health, providing assurance to respondents that their identities and information would be safe from disclosure even if requested by subpoena. The research was conducted between summer 2010 and spring 2011. The study received ethical approval from the Health Sciences and Behavioral Sciences Institutional Review Board of the University of Michigan.

### Online Survey

#### Participants

We used an online questionnaire to survey a convenience sample of 194 YMSM. To engage an ethnically diverse sample, we recruited via a variety of venues, eg, social media websites, dating websites, health clinics, bars/clubs, public postings, LGBT organizations, AIDS Service Organizations (ASOs). Participants in the individual interviews were also invited to complete the online survey.

#### Survey Procedures

Participants completed an online self-administered survey after indicating comprehension of the informed consent material and agreement to participate in the study. The survey was pilot-tested and was administered on a dedicated website using Sawtooth software. The survey took 30-45 minutes to complete. The overall survey was distributed over 108 screens with an average of 6 questions per screen; however, skip-response patterns were used, thus reducing survey length for most participants. The survey included questions regarding HIV/AIDS–related information acquisition and use patterns, gay community involvement, risk behaviors, and technology use. Participants were also able to save in-progress surveys and return later for completion. Participants did not have the opportunity to review their responses, and there were no completeness checks, prior to submission. Each participant received a $25 e-gift card for participating.

Web survey data were collected on a secure server under 128-bit SSL encryption and a firewall. After downloading, data were expunged from the server. To prevent multiple entries [42], we used participants’ email, IP address, browser/operating system, and time taken to complete survey to flag potential fraudulent/duplicative cases. We cross-checked email and IP addresses through web applications (eg, Facebook, IP lookup), without keeping this information or linking it to data. If verified, we treated a case as unique; otherwise, we did not use the entered data. We had 824 unique site visitors, as counted by unique IP address. We recorded 1034 survey entries, which included 194 eligible and complete cases, 16 incomplete entries, and 264 entries that were ineligible for study participation based on eligibility criteria. In addition, we detected 559 fraudulent entries, which were removed from our dataset. Our recruitment rate was 79.69% and, after excluding fraudulent cases, our completion rate was 92.38%. After verification, data were de-identified and transferred into SPSS software.

#### Survey Measures

##### Gay Community Involvement

We initially used an established, 17-item measure of gay community involvement [43]. When subjected to a principal axis factor analysis with varimax rotation, one factor was produced with an Eigenvalue of 5.13 and explained 31.273% of the variance. This factor was used to create an 5-item scale of gay community involvement, which included items such as, “In your opinion, do you feel that you are a part of the gay community in your area?”, “How many of your friends are men who also have sex with other men?”, “How much of your leisure time do you spend with men who also have sex with other men?” and “In a typical week, if you hang out with men who also have sex with other men, how much time do you spend at meetings or organizations?” This scale, which measured both behavioral and affective dimensions of community involvement, had good internal reliability (Cronbach alpha=.872).

##### Community to Which You Most Belong

To assess the possibility that YMSM had alternative community affiliations that might affect their HIV/AIDS-related HII, we asked participants to complete the following open-ended survey question, “People have different definitions for the term ‘community’. Thinking about the different communities that you belong to, please indicate below what is the community that you feel like you belong to the most.” Participants’ responses to this question were then content-analyzed by assigning emergent categories to these responses [44].

##### Social Costs of Information Seeking

Based on Chatman’s theory of information poverty and its insight regarding the potential social costs of information seeking in marginalized groups [2], we adapted an existing, 15-item scale regarding the social costs of information seeking in a workplace setting [45]. When subjected to a principal axis factor analysis with varimax rotation, one factor was produced with an Eigenvalue of 9.399 and explained 62.660% of the variance. A 5-item scale was then created that asked about participant agreement with the statement, “It would not be socially acceptable in my community to look for this information…”, with response options referring to HIV-related matters such as how to use a condom and where to obtain an HIV test. Responses were on a 5-point Likert scale (1=Strongly disagree, 5=Strongly agree). The scale has excellent internal reliability (Cronbach alpha=.965). Due to the high skewness of this variable, it was log-transformed for further analyses.

##### Community Relevance

Again, based on Chatman’s theory of information poverty [2], we created a 3-item scale that assessed the perceived relevance of HIV/AIDS information to one’s community. These items included: “HIV/AIDS is an important issue in my community” and “Men who have sex with men need to know everything they can about HIV/AIDS.” Responses were on a 5-point scale (1=Strongly disagree, 5=Strongly agree). Principal axis factor analysis with varimax rotation revealed a single factor with an Eigenvalue of 2.396 and explained 79.875% of the variance. The scale had high internal reliability (Cronbach alpha=.874).

##### Network Expertise Accessibility

This variable was calculated to refer to the availability of HIV/AIDS information from people close to the participant or those identified by the participant as people with whom they discussed “important personal matters,” including those with whom they have discussed or would feel comfortable discussing HIV/AIDS prevention and testing. After naming each network member, participants were asked to state whether they had ever discussed HIV/AIDS with that person and whether they considered that person “knowledgeable about HIV prevention.” Responses were on a 4-point scale (1=Completely disagree, 4=Completely agree). For each network member, an “expertise accessibility” multiplier variable was created for discussion of HIV/AIDS and the participant’s rating of that network member’s knowledge of HIV/AIDS. Then, a variable was created for “Total network expertise” accessibility, which summed the scores of expertise accessibility for all network members. Due to significant skewness, this variable was then log-transformed for statistical analyses.

##### Frequency of HIV/AIDS Information Seeking

This 1-item measure was adapted from the National Cancer Institute’s Health Information National Trends Survey (HINTS) [46]. The question asked: “In the past 12 months, how often have you looked for HIV/AIDS information from any source? By ‘source’ we mean people, organizations, documents, or the Internet.” Responses were on a 5-point scale (1=Never, 5=Very often).

##### Incidental Information Acquisition (IIA) Frequency

This 4-item scale was developed based on extant theory regarding non-purposeful information acquisition, including the role of an information-rich environment in facilitating such acquisition [26,47-49]. Responses were on a 4-point scale (1=Never, 4=A lot). A principal axis factor analysis with varimax rotation revealed that a single factor with an Eigenvalue of 2.39 explained 59.475% of the variance. Based on factor loadings, a final 3-item scale was created that included these items: “I accidently find information about HIV/AIDS while I look for information about other topics”, “I learn unexpected things about HIV/AIDS when I watch television or read the newspaper”, and “I learn unexpected things about HIV/AIDS when I talk to other people.” This scale had good reliability (Cronbach alpha=.798).

##### Frequency of Information Use

We developed an original 15-item scale that assessed use of HIV/AIDS information for a variety of topics relevant to HIV/AIDS risk and prevention. Principal axis factor analyses with varimax rotation showed that a single factor with an Eigenvalue of 8.962 explained 59.744% of the variance. Thus, a 10-item scale was created with responses to the question “In which of the following ways did you use the HIV/AIDS information that you got in the past 12 months? Did you use the information to...”. Options included finding a place to get tested for HIV, deciding whether to ask a partner his HIV status, deciding whether to get tested for HIV, and deciding whether to ask a partner to obtain an HIV test. Responses were on a 5-point scale (1=Never, 5=A great deal). The scale had excellent reliability (Cronbach alpha=.937).

##### Technology Access

Participants were asked whether or not they have technologies that may provide Internet access, including desktop/laptop computers, cell phone/smartphone, PDA, e-readers, music players, and game consoles.

##### Internet Use Levels

Participants were asked how often they use the Internet at a variety of locations. Options included home, school, work, public library or community center, mobile device, or other. The response scale varied from “Less often than every few weeks or never” to “Several times a day”. Due to high levels of Internet use in the sample, binary variables were then created across all Internet access locations to note whether the participant “Uses the Internet several times a day” or “Uses the Internet less than several times a day”.

##### Technology-Mediated Personal Network Member Communication

As mentioned, participants were asked to specify up to 7 people with whom they discussed “important personal matters”. Each participant was asked how often they communicate weekly with each of these named network members using the Internet, phone (not including texting), or face to face. They were also asked how many texts they sent per day to that person. Daily texts were then transformed into a weekly value. Following this, the proportion of overall daily contacts with each network member through each communication medium was calculated. This number was then used to calculate an overall average for each communication media for each participant across all of their network members.

##### Personal Network Members Met Online

Participants were asked how they met each of their network members. Response options included family, school, social gathering/through friends, online, work, and other. A binary variable was created to indicate whether a network member was met online. The total number of network members whom the participant had met online was then calculated. Because this was a highly skewed variable, this number was transformed into a binary variable for each participant for whether or not he had met any network members online.

##### Internet Use For Online Dating

Participants were asked how many times in the past 2 months they had used the Internet to: 1) find someone to date, or 2) to “hook up” (ie, have a sexual encounter). The 7-point response scale ranged from “Never” to “More than once a day”. Because these variables were skewed, a binary variable was created to reflect whether or not the person had used the Internet for either purpose in the past 2 months.

##### Time Spent Chatting With Other MSM Online

Participants were asked how much time they spend hanging out with other MSM by “chatting on the Internet”. The 4-point response scale ranged from 1=Not at all to 4=More than 10 hours.

##### Online HIV/AIDS Information Seeking Frequency

Participants were asked how much they had used three online source types to obtain HIV/AIDS information in the past 12 months. Options included “Internet sites for men who have sex with men”, “Social networking sites (like Facebook or Twitter)”, and “All other Internet sites”. The 4-point response scale ranged from 1=Never to 4=Often. A principal axis factor analysis with varimax rotation was conducted, producing one factor with an Eigenvalue of 2.52 that explained 75.075% of the variance. Values on this new scale were skewed, and therefore, were classified as never, often, or rarely/occasionally using any online HIV/AIDS information source.

##### Demographic Covariates

Participants were asked to state their age, race (White/European American, Black/African American, Asian, Native American/Alaska Native, Hawaiian/Pacific Islander and Other), ethnicity (Hispanic/Latino or not), sexual identity (gay/bisexual/heterosexual), and highest level of education completed. Due to the disparity between whites and African-American and Latino MSM in new HIV infections, a binary “minority” variable was created for African Americans and Latinos. Due to the distribution of the education variables, we also created a binary education variable to indicate whether the participant had a high school education or an education beyond high school.

#### Statistical Analysis of Survey Data

We calculated descriptive statistics about the respondents’ gay community involvement, categories for the community to which they most belong, HII, technology use, and demographics. We then tested multiple linear regression models that took each of the key community- and information-related variables as the dependent variables. The independent variables in these models included community- and information-related variables, as well as demographic covariates. Assumptions for multiple linear regressions were met. Skewness and kurtosis values for the dependent and independent variables were within range for normality, and residuals plots and partial plots looked acceptable. Lack of multicollinearity among the predictors was indicated by all Pearson’s correlation measures being < 0.7, variance inflation factor values < 10, and tolerance values > 0.10. Cook’s *d* values were well below 10, so no outliers affected the results. Once the initial regressions were conducted, those results were used to determine which paths to include in a final model. Structural equation modeling software (SPSS Amos, version 20) was used to perform recursive path analysis with observed variables and to estimate the model. Because of power considerations, sample size did not allow for reliable testing of model fit; therefore, fit statistics are reported only briefly in the analyses.

### Qualitative Interviews

#### Interview Participants

Due to the modest levels of variance in HII predicted by our regression models (*R* ^*2*^=0.07-0.28), we conducted a focused analysis of interview data to identify additional gay community involvement factors that may help explain HIV/AIDS-related HII among YMSM (RQ1). Our initial interview sample included 29 YMSM who were recruited via social media websites, dating websites, health clinics, bars/clubs, public postings, LGBT organizations and ASOs. To permit in-depth examination of our research question, we theoretically sampled [50] a subset of 19 interview participants (drawn from the original 29) with the highest levels of gay community involvement, as determined by perceptions of belonging to their local gay community, involvement in gay-related organizations, and the prevalence of other MSM in their social networks. Seven of these participants were current or previous volunteers or paid staff for HIV prevention initiatives sponsored by LGBT organizations and/or ASOs.

#### Interview Procedures

In-depth, semi-structured interviews [51] were conducted using an interview guide with open-ended questions, follow-up questions, and probes [52]. The interviews focused on participants’ perceptions of community, their HIV/AIDS-related HII, and their HIV testing decisions and experiences. Interviews lasted from 45 to 90 minutes. Interviews were audio-recorded and transcribed. Participants’ social networks were elicited with name-generator questions [53], followed by visualization exercises [54] that gathered data about network structure and demographics. Each participant received a $30 gift card for participating.

#### Qualitative Analysis of Interviews

We conducted a grounded theory [50] analysis of interview transcripts using the constant comparison method [55]. Initially, we conducted open coding [50] using gerunds so as to focus on actions and processes [56], followed by axial coding [50] in order to define conditions, actions/interactions, and consequences associated with our emergent core category. Selective coding [50] and memoing were also pursued to further define and interrogate this category [56].

## Results

### Participant Characteristics

Survey participants’ average age was 20.66 (see Table 1). More than half of the sample (57.2%) was Black/African American, and 18% of participants were Hispanic/Latino. Approximately half of the sample (52.6%) had a high school education or less. The majority identified as gay (84.5%), with 13.5% identifying as bisexual. Fifteen participants (11.6%) reported that they had received an HIV-positive test result.

The majority felt that the community to which they most belonged was the Gay/Queer/LGBT community (65.8%), with the next most common response being none (10.2%). Several YMSM defined their primary community as smaller subgroups of people united around alternative principles, such as shared values (3.7%) or friendship/kinship (4.8%). However, it is likely that these groups included other MSM, since 4 (25%) of the participants who chose these smaller subgroups also indicated that “some” or “all” of their friends were MSM, and 8 (50%) stated that “a few” were. A minority of participants (15.5%) “most belonged to” an alternative social group. The most frequently named alternative social groups were school/workplace (7.0%), city/neighborhood (2.1%), style/fashion subculture (2.1%), sports/recreation (1.6%), ethnic/cultural group (1.6%), and churches (1.1%). There was a large association [57] between naming Gay/Queer/LGBT as one’s key community and our aforementioned measure of gay community involvement (η=0.519; CI 0.414-0.648).

As might be expected with a web survey sample, participants were heavy Internet users, with 89.7% of respondents using the Internet several times a day (see Table 2). Participants also had significant access to technological devices—100% of participants had access to at least one. Of these, 80.5% had a cell phone, 65.6% an iPod/MP3 player, 61.5% a laptop computer, and roughly half (53.2%) a game console. As for uses of technology, participants indicated that an average of 43% of their weekly contacts with their close network members were through texts. An average of 12% of interactions took place on the Internet, 12% were on the telephone, and 19% were face to face. Although just over a third of the participants had used the Internet to meet other men for dating or sex in the past 2 months, only 13.4% said that they had met one of the people that they discuss “important personal matters with” online. A large proportion (41.9%) did not spend any time chatting with other MSM online in a typical week. At the same time, 33.5% of respondents said that they spent 3 hours or more per week doing so. Despite their significant Internet usage, a small proportion (7.7%) had frequently obtained HIV/AIDS information online in the past year, and 58.7% of all participants had looked for HIV/AIDS information online at least rarely in the past 12 months. However, 31.3% indicated that they had not done so at all in that time. The most popular online source for HIV/AIDS information was Internet sites for MSM: 100 participants (51.5%) had used this source at least rarely over the previous year.

Like the survey participants, the mean interview participant age was just under 21, and the majority was African American and gay-identified (see Table 3). A similar proportion of the samples was also Hispanic/Latino (17.5% of survey participants vs. 15.8% of interviewees). A small minority of both samples were HIV-positive.

### Survey Results

#### Prediction of Community and Information-Related Variables

Table 4 shows that gay community involvement was not associated with demographic covariates, including age, education, or minority status. Similarly, minority status was independent of gay identity (χ^2^(1)=.019; *P*=.890). In support of Hypothesis 1, gay community involvement was associated with significantly lower perceived social costs of information seeking. The covariate of having more than a high school education was associated with more perceived social costs. Overall, however, only a small proportion (9%) of the variance in social costs was explained by gay community involvement and education, with most of the variance (7%) explained by community involvement.

In support of Hypothesis 2, gay community involvement and social costs had significant associations with access to HIV/AIDS expertise in personal networks. On an unadjusted basis, participants with more education and those who were racial/ethnic minorities had less access to HIV/AIDS expertise in their networks, but these effects disappeared after adjustment for community involvement and social costs. 14% of the overall variance in network expertise was accounted for in the final model.

Community relevance was predicted on an unadjusted basis by community involvement, social costs, and network expertise access, although it was not predicated on any demographic covariates. However, the final model, which accounted for 14% of the variance in community relevance, included only community involvement as a significant predictor. Thus, Hypothesis 3 was supported.

Table 5 shows that, in accordance with Hypothesis 4, gay community involvement was a significant predictor of incidental information acquisition (IIA), both before and after adjustment. Sixteen percent (16%) of the variance in IIA was explained by community involvement. Younger men and those with more education reported more IIA, but these effects disappeared after adjustment. Similarly, a marginally significant relationship between community relevance and IIA disappeared after adjustment.

Hypothesis 5 also received support. Those with greater gay community involvement had sought HIV/AIDS information more frequently than those with less involvement. Social costs of information seeking, community relevance, and IIA were all significant predictors of information seeking frequency on an unadjusted basis. However, each of these effects disappeared in the full regression model, leaving only community involvement as a significant gay community-related predictor. This result meant that Hypothesis 7 was unsupported, since social costs and community relevance could not act as mediators between community involvement and information seeking without these variables having a direct association with information seeking. As for covariates, minority men sought HIV/AIDS information more frequently than whites; this variable was significant in the final model, although its contribution to prediction was smaller than community involvement (*R* ^2^ change=5% of the variance in information seeking). For information-seeking frequency, 9% of the variance was predicted by such gay community involvement alone (see Figure 1).

The most robust regression model sought to predict HIV/AIDS information use, with 28% of the variance in the model explained by included variables: community relevance, network expertise access, and both IIA and information seeking. Therefore, Hypothesis 6 was supported. The magnitude of effect for community relevance (path coefficient=.273) was comparable to that for incidental information acquisition and seeking (path coefficients=.215 and .284, respectively). Significant direct effects for information use disappeared once adjusted for its mediators. Thus, all effects for community involvement on information use were indirect, providing support for Hypothesis 8 (see Table 6).

Therefore, overall, four community-related variables were significant in predicting the amount of information acquisition and/or use: 1) community involvement, 2) social costs of information seeking, 3) network expertise accessibility, and 4) community relevance. The final path model predicted 28% of the variance in information use, 14% of the variance in incidental information acquisition, and 9% of the variance in information seeking (see Figure 1). Furthermore, without the effects of information acquisition on information use, the variables of community involvement, community relevance, and network expertise access alone explain 17% of the variance in information use.

#### Model of Community Involvement Factors in Human-Information Interaction

A recursive path analysis with observed variables was estimated with AMOS structural equation modeling software version 19. The resulting model is depicted in Figure 1. Table 6 contains coefficients for direct and indirect effects. Demographic covariates (education, race) offered little improvement in the prediction of HII dependent variables, and thus, they were excluded from the final model. The final model showed good fit: the root mean square error of approximation (RMSEA) was 0.054 (90% CI 0.000-0.101), the Chi-square was non-significant (χ^2^(11)=17.105; *P*=.105), and the overall Comparative Fit Index (CFI) was 0.967.

#### Technology, Community, and Information Interaction

Hypothesis 9 received partial support. Significant positive relationships exist between gay community involvement and use of the Internet at least several times a day (*r*_pb_=0.153, *P*=.040) and online information seeking regarding HIV/AIDS (*r*=.302, *P*=<.001). However, a significant negative relationship exists between gay community involvement and hours spent chatting with other MSM on the Internet (*r*=-.175, *P*=.018). In addition, no significant relationships exist between gay community involvement and the proportion of network contacts via texting (*r*=-.080, *P*=.456) or the Internet (*r*=-.152, *P*=.108). Furthermore, there were no significant relationships between gay community involvement and online dating (*r*_pb_=-0.113, *P*=.129) or having met at least one close network member online (*r*_pb_=-0.047, *P*=.531).

### Interview Results

Due to the modest predictive power of the existing model for HII-related dependent variables, we sought to refine our model by investigating what additional gay community-related factors, if any, may help to explain HIV/AIDS–related HII among YMSM (RQ1). Our grounded theory analysis of interview transcripts yielded a key category: *information sharing.* Analyses showed that the conditions that facilitated information sharing were YMSMs’ endorsement of *enacting pro-social community values* (see Table 7). Accordingly, 9 YMSM defined community as *looking out for each other*, particularly when under some form of attack or threat, and an additional 9 understood community as *working together,* or striving for common goals. Because HIV/AIDS was seen to be a serious threat to the community (*community relevance*), YMSM sought to enact their pro-social community values by *making a difference* in reducing the burden of HIV/AIDS in their community. A key aspect of making a difference for these YMSM was *informing community;* indeed, some youth believed that information sharing was a key characteristic of “community” as a value.

*Information sharing* included the following key actions/interactions: *organizing events,* such as community discussions or video screenings*; disseminating messages* through flyers, t-shirts, workshops or other media; *encouraging safety* through interpersonal discussions with friends and acquaintances; and *referring and recommending,* so as to connect friends with HIV testing sites or other help sources.

A consequence of information sharing was *interacting with more information*. Indeed, information sharing emerged as a potential correlate of all HII variables included in our model. For example, information sharing motivated *information seeking* about the disease, since one needed to acquire information before sharing it. This information seeking often involved longer-term activities such as attending HIV/AIDS–related workshop series, internships or training, as well as episodic activities such as Internet searching and asking questions. Sharing information also comingled with efforts towards *countering stigmatization* of both HIV/AIDS–related help seeking and PHAs—which could ultimately affect perceived *social costs of information seeking.* Furthermore, information sharing—especially if formalized through volunteer or paid work with LGBT organization or ASOs—often placed YMSM in information-rich environments that facilitated ongoing *incidental information acquisition.* Information sharing efforts also led participants into contact with other people who were knowledgeable about HIV/AIDS, especially other volunteers or coworkers. Such *network expertise accessibility* meant that participants had many network-mediated opportunities [27] for asking questions about HIV/AIDS (information seeking). Furthermore, YMSM made a strong connection between information sharing and their *information use* for making decisions about their sexual health. In particular, sharing information appeared to increase participants’ personal motivation for safer sex and HIV testing and their associated commitment to acting as role models for others.

**Table 1:** Survey participant demographics (n=194).

		Number	Valid Percent
**Age, mean (SD)**		20.66 (1.71)	
**Race** ^**a**^			
	Black/African American	111	57.2
	White/European American	75	38.7
	Native American/Native Hawaiian/Pacific Islander	10	5.2
	Asian	12	6.2
	Other	13	6.7
**Hispanic/Latino**		34	17.5
**Education**			
	Some high school	10	5.2
	High school/GED	92	47.4
	Technical school	3	1.5
	Some college	69	35.6
	Bachelor’s/graduate degree	18	9.8
**Sexual identity** ^**a**^			
	Gay	154	84.5
	Bisexual	26	13.5
	Heterosexual	5	3.6
	Other	6	3.1
**HIV-positive**		15	11.6
**Primary community membership**			
	Gay/Queer/LGBT	123	65.8
	None	19	10.2
	School/Workplace	13	7.0
	Family/friends	9	4.8
	Values-based community (eg, communication, love, togetherness, beauty)	7	3.7
	City/neighborhood	4	2.1
	Style/fashion (eg, urban prep, stoner)	4	2.1
	Sports/recreation	3	1.6
	Ethnic/cultural group	3	1.6
	Church	2	1.1

^a^ More than one response possible.

**Table 2:** Survey participants’ technology use and information interaction (n=194).

		Number	Valid Percent
**Technology access**			
	Desktop computer	71	36.8
	Laptop computer	118	61.5
	Cell phone (including smart phones such as iPhone, Android, BlackBerry or similar device)	153	80.5
	PDA or personal data device	17	8.9
	E-reader (eg, Kindle, iPad)	36	18.6
	iPod or MP3 player	126	65.6
	Game console (eg, Xbox, Playstation)	100	53.2
**Internet use levels**			
	Several times a day	174	89.7
	At least once a day	18	9.3
	Less than once a day	2	1
**Personal network member communication**			
	Mean proportion on Internet (SD)	0.12 (0.13)	
	Mean proportion on texting (SD)	0.43 (0.19)	
	Mean proportion on phone (not including texting) (SD)	0.12 (0.11)	
	Mean proportion on face-to-face (SD)	0.19 (0.15)	
**Met at least one personal network member online**		24	12.4
**Internet use for online dating**		70	36.5
**Time spent chatting with other MSM online per week**			
	More than 10 hours	27	14.1
	3-10 hours	37	19.4
	Up to 3 hours	47	24.6
	Not at all	80	41.9
**HIV/AIDS information seeking frequency – all sources**			
	Very often	27	13.9
	Often	31	16.0
	Sometimes	72	37.1
	Rarely	37	19.1
	Never	27	13.9
**Online HIV/AIDS information seeking frequency (n=166)**			
	Often	15	7.7
	Occasionally or rarely	99	59.6
	Never	52	31.3

**Table 3:** Interview participant demographics (n=19).

		Number	Valid Percent
**Age, mean (SD)**		20.79 (1.96)	
**Race ^a^**			
	Black/African American	12	63.2
	White/European American	4	21.1
	Native American/Native Hawaiian/Pacific Islander	2	10.5
	Asian	1	5.3
**Hispanic/Latino**		3	15.8
**Sexual identity^a^**			
	Gay	13	68.4
	Bisexual	6	31.6
**HIV-positive**		1	5.3

^a^ More than one response possible.

**Table 4:** Linear regressions for community-related variables.

Independent variable		Dependent variable							
		Gay community involvement (GCI)		Social costs of HIV/AIDS information seeking		Network access to HIV/AIDS expertise		Community relevance of HIV/AIDS information	
		Beta	*P*	Beta	*P*	Beta	*P*	Beta	*P*
Age	Unadj.	–.070	.346	–.103	.154	.079	.272	.011	.881
	Adj.	—	—	—	—	—	—	—	—
Education level	Unadj.	.001	.988	.163	.023	–.269	<.001	–.083	.256
	Adj.	—	—	.164	.022	–.057	.422	—	—
Racial /ethnic minority	Unadj.	.064	.393	–.075	.296	–.173	.016	–.003	.968
	Adj.	—	—	—	—	–.031	.654	—	—
Gay community involvement (GCI)	Unadj.	—	—	–.272	<.001	.271	<.001	.356	<.001
	Adj.	—	—	–.272	<.001	.318	<.001	.303	<.001
Social costs of HIV/AIDS information seeking	Unadj.	—	—	—	—	–.345	<.001	–.242	.001
	Adj.	—	—	—	—	–.149	.044	–.142	.062
Network access to HIV/AIDS expertise	Unadj.	—	—	—	—	—	—	.184	.011
	Adj.	—	—	—	—	—	—	.053	.485
Community relevance of HIV/AIDS information	Unadj.								
	Adj.								
Incidental HIV/AIDS information acquisition frequency	Unadj.	—	—	—	—	—	—	—	—
	Adj.	—	—	—	—	—	—	—	—
Frequency of HIV/AIDS information seeking	Unadj.	—	—	—	—	—	—	—	—
	Adj.	—	—	—	—	—	—	—	—
R^2^ adjusted		—		.091		.135		.139	

**Table 5:** Linear regressions for information interaction variables.

Independent variable		Dependent variable					
		Incidental HIV/AIDS information acquisition frequency		Frequency of HIV/AIDS information seeking		HIV/AIDS information use for decision making	
		Beta	*P*	Beta	*P*	Beta	*P*
Age	Unadj.	–.130	.070	.065	.368	–.026	.724
	Adj.	–.031	.686	—	—	—	—
Education level	Unadj.	.180	.012	.001	.984	.008	.913
	Adj.	.168	.030	–	–	–	–
Racial /ethnic minority	Unadj.	–.030	.675	.234	.001	.092	.200
	Adj.	—	—	.226	.001	—	—
Gay community involvement (GCI)	Unadj.	.378	<.001	.301	<.001	.257	<.001
	Adj.	.368	<.001	.192	.020	–.059	.448
Social costs of HIV/AIDS information seeking	Unadj.	.012	.868	–.194	.007	–.108	.135
	Adj.	—	—	–.100	.175	—	—
Network access to HIV/AIDS expertise	Unadj.	–.014	.850	.071	.324	.244	.001
	Adj.	—	—	—	—	.192	.005
Community relevance of HIV/AIDS information	Unadj.	.137	.058	.206	.004	.375	<.001
	Adj.	.020	.784	.103	.173	.273	<.001
Incidental HIV/AIDS information acquisition frequency	Unadj.	—	—	.162	.024	.274	<.001
	Adj.	—	—	.083	.274	.215	.002
Frequency of HIV/AIDS information seeking	Unadj.	—	—	—	—	.371	<.001
	Adj.	—	—	—	—	.284	<.001
R^2^ adjusted		.157		.143		.278	

**Table 6:** Standardized total, direct and indirect path coefficients for model (see Figure 1) (N=194).

	Standardized Total effects			Standardized Direct effects			Standardized Indirect effects		
Parameter estimate	Est.^a^	CI	*P*	Est.	CI	*P*	Est.	CI	*P*
GCI – Social costs of information seeking	–.272	(–.358 - –.138)	.032	–.272	(–.358 - –.138)	.032	—	—	—
GCI – Network expertise access	.271	(.141-.388)	.011	.271	(.141-.388)	.011	—	—	—
GCI – Community relevance	.356	(.246-.463)	.009	.356	(.246-.463)	.009	—	—	—
GCI – Incidental information acquisition	.378	(.257-.476)	.011	.378	(.257-.476)	.011	—	—	—
GCI – Information seeking frequency	.301	(.174-.382)	.018	.301	(.174-.382)	.018	—	—	—
GCI – Information use	.300	(.234-.361)	.013	—	—	—	.300	(.234-.361)	.013
Social costs of information seeking – Network expertise access	–.293	(–.386 - –.173)	.020	–.293	(–.386 - –.173)	.020	—	—	—
Social costs of information seeking – Information use	–.053	(–.095 - –.025)	.007	—	—	—	–.053	(–.095 - –.025)	.007
Network expertise access – Information use for decision making	.181	(.097-.273)	.008	.181	(.097-.273)	.008	—	—	—
Community relevance – Information use for decision making	.261	(.160-.386)	.005	.261	(.160-.386)	.005	—	—	—
Incidental information acquisition –Information use for decision making	.198	(.072-.285)	.021	.198	(.072-.285)	.021	—	—	—
Information seeking frequency –Information use for decision making	.276	(.169-.373)	.012	.276	(.169-.373)	.012	—	—	—

^a^ Est. = estimate.

**Table 7:** HIV/AIDS information sharing.

Categories	Concepts	Sample participant quotations
**Conditions: Enacting pro-social community values**		
	Looking out for each other	“*A group of people who look out for one another, nurture each other, fight for each other…”* * “…having somebody’s back…”*
	Working together	“*I hear the word ‘community’, I hear unity in it, so that means everyone must come together to be one unit…everyone working together equally, picking up the slack …”* * “…a group of people that… try to do anything that will work for…the common good for the group...”*
	Making a difference	“*HIV and AIDS…I grew a passion for it…knowing that it was something out there that was destroying the community…I…can have a big impact on…protecting people from [it].”*
	Informing community	“…*when I hear ‘community’…that brings to my head friends, family helping out each other and informing each other about certain things and having each other’s backs.”* * “…when…I talk about safe sex with people, that’s...my community…”*
**Actions/Interactions: Information sharing**		
	Organizing events	“…*a lot of people…were starting to get infected and… your heart hurt… …it just led to this…urgent need to talk to us… …[so we organized] a community discussion…because it seems like we’d get the information, then it dies…”*
	Disseminating messages	“…*mostly…the gay community has it and people have died from it … made me feel sad, and [I] wanted… help… I use to volunteer…like do flyers…”*
	Encouraging safety	“…*if you are around me, I’m gonna pull you in, like ‘…just protect yourself …that’s your body’…talking to people…”*
	Referring and recommending	“…*I send them random texts, send them Facebook messages, ‘have you gotten tested yet?’…‘do you want to go out tonight? Yeah, let’s go get tested [for HIV]…’*
**Consequences of information sharing: Interacting with more information**		
	Information seeking	“*...learning this information and being able to put it back into the community. It’s kind of my purpose…”*
	Countering stigmatization and the social costs of information seeking	“…*if they see somebody, they be like ‘ok well I just met this person but he have HIV so I’m not going to talk to him’…I try to get them out of that…”*
	Incidental information acquisition	“*I know the signs, symptoms… how to test people… by watching, paying attention, going through it, trying to help out…if you volunteer at enough places you’ll get the knowledge of [HIV/AIDS]…”*
	Network expertise accessibility	“*I had, the fortune of having a friend who was HIV positive and so he told me about his viral count and having to manage his medications and…health… that’s where I got a lot of my information…”*
	Information use	“… *how can I tell one person or teach a person of how to protect themselves when I’m not doing it myself? So it put me on my Ps and Qs…more about safer sex…”* * “…before I joined that [HIV prevention program], I didn’t count oral sex as sex, I didn’t use…condoms…”*

## Discussion

Results of this study support our central premise that HIV/AIDS information interaction and gay community involvement are related among YMSM. Gay community involvement was a significant predictor of all HII-related variables included in the study: social costs, community relevance, network expertise access, incidental information acquisition, information seeking, and information use. The overall model also predicted a non-trivial, although modest, amount of the variance in information acquisition frequency (9-14%) and information use (28%). Moreover, community-related variables alone explained 17% of the variance in information use. Community-related variables were also stronger predictors of HII than demographics. Furthermore, our data offer insight into *how* community matters: YMSM who are more involved in the gay community acquire more HIV/AIDS information, see that information as more relevant, and have more knowledgeable, close network members with whom they may discuss that information. Each of these factors appear to contribute to HIV/AIDS information use. People who are more involved in the gay community also perceive fewer social costs in relation to looking for HIV/AIDS–related information, which correlates with more access to knowledgeable people in one’s network. Qualitative findings also suggest that community involvement may be related to enacting pro-social community values through information sharing. In turn, information sharing may be associated with each of the other HII variables included in the model. Overall Internet use and online HIV/AIDS–related information seeking were also correlated with gay community involvement, and Internet sites for MSM were the most used online information sources. However, some aspects of the role of technologies in the community-HII relationships are ambiguous, since some technology uses were related to community involvement, one social use was negatively related, and some social uses had no relationship at all.

Our findings suggest that HIV/AIDS-related HII and associated technology uses are community-embedded processes, yet the majority of HIV/AIDS-related informatics interventions to date attempt to influence individual-level constructs, such as knowledge, attitudes, and self-efficacy [58]. Results suggest that this approach, while valuable, may be insufficient because it does not account for the social contexts of information acquisition and use. Thus, existing interventions may not be positioned to account for differential reception of interventions within communities, and unplanned uses and effects. Indeed, our related work shows that when they are available within high-prevalence communities, technologies may be incorporated into HIV/AIDS-related communication processes in surprising ways [59]. Moreover, clinically oriented health informatics research documents the unplanned consequences of health information technology deployed in clinical settings (eg, [60,61]). Attention to the community-embedded nature of HIV/AIDS information and technologies may help us to more effectively conceptualize, design, and deploy informatics interventions that respond to the unique needs and characteristics of different groups. Additionally, by focusing primarily on individuals, informatics interventions miss the potential for community-level intervention and effects. They also do not consider the potential importance of pro-social information sharing and the potential for promoting information sharing through social media, texting, and other technologies. However, the community informatics field has shown the potential for technologies to be used to develop local social networks and facilitate collective action [62-65]. Given that offline activism and volunteering may be effective community-based HIV/AIDS prevention strategies [66], our research suggests that we may benefit from considering how informatics interventions can also be designed as community-level interventions and vice versa.

Our model is strengthened by inclusion of theoretical mediators that help explain the effect of community involvement on information acquisition and use. Therefore, we offer the first quantitative assessment of important concepts that have emerged from qualitative field work in information science, such as social costs of information seeking and collective relevance (eg, [2,67]). Moreover, this is one of the first studies to highlight information sharing as a potentially important form of community-embedded HII. Such confirmation and extension help answer calls for increased insight into information production, acquisition, and use in everyday life [1,4,68]. Moreover, this model suggests potential bases for community-level interventions. For example, the model’s mediators suggest that gay community involvement provides two resources that may be critical for the use of acquired HIV/AIDS information: 1) valuing of that information through a belief that it is relevant to one’s group, and 2) supportive and knowledgeable network members with whom one can talk about HIV/AIDS. This finding opens previously unrealized possibilities for both public health and informatics interventions, such as potentially providing community-based services that help MSM understand the relevance of HIV/AIDS information and support them in discussing HIV/AIDS information with knowledgeable people whom they trust. Our research also suggests, as we have argued elsewhere, that stigma-reduction interventions may improve access to HIV/AIDS information in communities [3]. We also highlight the fact that interventions that engage at-risk groups in preventing HIV/AIDS among their members (eg, [69]) may have under-acknowledged consequences for information sharing in a variety of forms. In these senses, we advocate broadening the public health field’s conception of community-level HIV/AIDS interventions to highlight information interaction as a focus for intervention, as well as a desired outcome of our efforts. In an era of reduced funding, current and future mediators included in this model may prove to be especially valuable outcome measures for community-level interventions within the context of public health practice.

Our findings raise questions about the potential role of information interaction in observed relationships between gay community involvement and HIV risk behavior. MSM who are more involved in the community have more sexual partners [34], particularly if they frequent gay bars/clubs [70]. Greater attendance at gay bars/clubs is also correlated with more high risk sexual behavior [14,70,71], partly due to its association with number of sexual partners [70] and exposure to alcohol and other drugs [35]. However, involvement in other gay community activities, such as sports teams, gay organizations, ASOs, and political activism may be protective [25,66,72,73]. An Australian study showed that HIV testing among MSM was associated with having more gay friends [74]. One study and a theoretical model suggest that such protective effects may be linked to the effect of community involvement on safer sex self-efficacy [66,70]. Researchers also posit that a protective effect for ASO involvement may be linked to positive peer norms regarding condom use, more positive self-identity, and lower levels of alienation [66]. Despite these observed correlations, we know little about potential mechanisms that may underlie such community involvement-risk behavior associations [70]. Our results therefore generate a novel, information-based hypothesis at the community involvement-HIV risk nexus. The next step in investigating potential associations is to establish a connection between community-embedded information interactions and risk behavior. While such a connection largely remains to be demonstrated, promising study results reported elsewhere show that information acquisition and use are significant predictors of MSMs’ intentions to seek HIV testing [75]. Our qualitative results also suggest a potential association between information sharing and use of information to make sexual health-related decisions. Further research within larger samples is needed to rigorously assess these potential associations.

While our research focuses on YMSM and gay community involvement, our findings may have relevance for other illnesses and community contexts, since prior research in other contexts has shown that communities may vary widely with regard to media and community organization involvement in health communication [76]. Furthermore, there is varied health knowledge in different communities [77-79]. Geographic communities experiencing health disparities may also have a higher prevalence of ambient, negative health messages [80] and have fewer exposures to positive health promotion messages [81]. One study also revealed that one’s participation in health communication activities in one’s local community is linked to understanding how to prevent illnesses prevalent in that community [82]. Although suggestive, further research is needed to determine whether the relationships included in our HIV/AIDS-related model hold in such varied health and community contexts and with what effects.

The overall finding that YMSM with greater involvement in the gay community used the Internet more resonates with research conducted in the general adult American population. Internet communication facilitates maintenance of a wide range of geographically dispersed relationships [83] that seamlessly shift between different communication media. The popularity of texting to communicate with participants’ close network members is also in concordance with this prior research [83]. However, results showing that those who spend more time chatting with other MSM online were less involved in the gay community were unexpected. Specific types of online activities may have an impact on friendship formation and feeling a part of the community. On the other hand, given that our measure of gay community involvement included time spent at meetings and organizations, there may be a simple time tradeoff at play, with people who spend a great deal of time chatting online having less time to devote to such organizational involvements. Nevertheless, this could mean that different technologically mediated strategies for community building among MSM would differ in effectiveness. Such possibilities merit further investigation.

### Limitations

Although the purpose of the study was to identify whether and how much community involvement predicted human-information acquisition, the overall magnitude of prediction for information seeking and incidental acquisition were relatively low (*R* ^2^ =9% and 15%, respectively). Although better for information use (28%), the magnitude remains modest. Following a tradition of research in information science (eg, [84]), further variables concerning the user’s situation may offer additional explanatory power. Furthermore, one may argue that YMSM may have had multiple community affiliations and that these affiliations could have confounded relationships at the gay community-HII nexus. However, participants were given the opportunity to name the community to which they most belonged, with the majority (65.8%) specifying the Gay/Queer/LGBT community and a minority (15.5%) identifying alternative communities—each of which lacked the historical and present burden of HIV/AIDS that is found in the Gay/Queer/LGBT community. Therefore, we do not expect that alternative community affiliations would be an important predictor of HIV/AIDS-related HII in our sample. Due to limited power based on the small sample size (n=194) and the pre-selection of variables, model fit statistics should be interpreted with caution. Finally, further research is needed through offline survey modes with men of all ages and in other geographical areas to assess the generalizability of this study’s findings to the larger MSM community and to assess the potential place of information sharing in a refined model. Furthermore, the model currently focuses specifically on the case of MSM and HIV/AIDS; applicability to other communities and diseases awaits verification.

### Conclusion

This research showed that, in a web-based sample of young MSM, gay community involvement was a significant predictor of a series of HIV/AIDS–related information interaction and technology use variables. Moreover, our model demonstrated that greater information use was predicted by social costs of information seeking, perceived community relevance, and network expertise accessibility. We also highlight the potential importance of a new variable, information sharing, at the community-HII nexus. Our findings suggested partial support for our hypothesis that YMSM who were more involved in the gay community would make heavier use of technologies to socialize with others. Together, these findings suggest that HIV/AIDS information interaction and technology use should be conceptualized as community-embedded processes as well as individual ones. Such recognition highlights the potential for novel, community-level health informatics interventions, while allowing us to perceive informational dynamics underlying community life that we did not see before.

## Abbreviations

ASO: AIDS Service Organization
CHI: consumer health informatics
GCI: gay community involvement
HII: human-information interaction
IIA: incidental HIV/AIDS information acquisition
IT: information technology
MSM: Men who have sex with men
PHA: people with HIV/AIDS
YMSM: young men who have sex with men
